# Injectable Thermosensitive Nanocomposites Based on Poly(*N*-vinylcaprolactam) and Silica Particles for Localized Release of Hydrophilic and Hydrophobic Drugs

**DOI:** 10.1021/acs.langmuir.2c03160

**Published:** 2023-02-06

**Authors:** Lucas S. Ribeiro, Renata L. Sala, Thaiane A. Robeldo, Ricardo C. Borra, Emerson R. Camargo

**Affiliations:** † Interdisciplinary Laboratory of Electrochemistry and Ceramics (LIEC), Departament of Chemistry, Federal University of São Carlos (UFSCar), Rod. Washington Luis km 235, CP 676 São Carlos, São Paulo 13565-905, Brazil; ‡ Laboratory of Applied Immunology, Federal University of São Carlos (UFSCar), São Carlos, Rod. Washington Luis km 235, CP 676 São Carlos, São Paulo 13565-905, Brazil

## Abstract

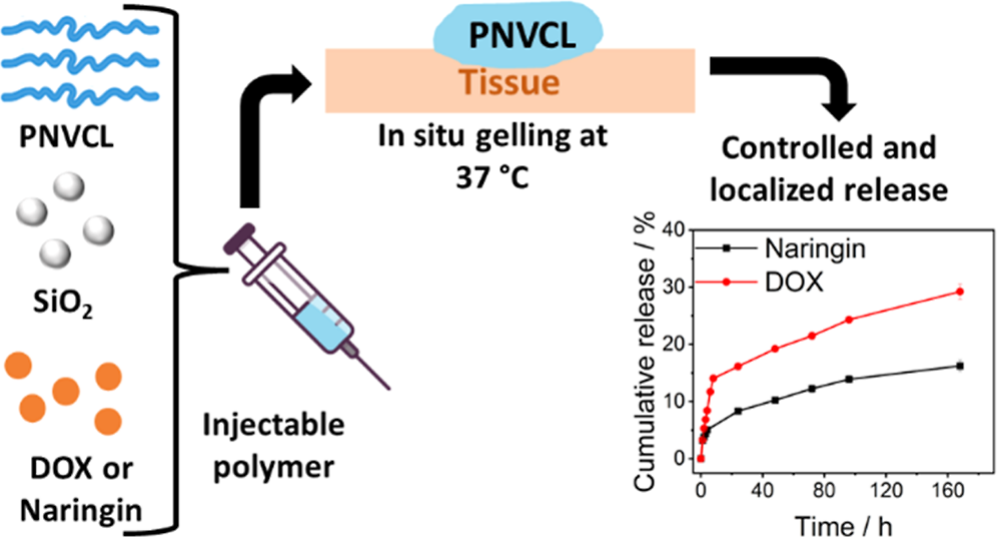

The systemic delivery of drugs employed by conventional methods has shown to be less effective than a localized delivery system. Many drugs have the effectiveness reduced by fast clearance, increasing the amount required for an efficient treatment. One way to overcome this drawback is through the use of thermoresponsive polymers that undergo a sol–gel transition at physiological temperature, allowing their injection directly in the desired site. In this work, thermosensitive nanocomposites based on poly(*N*-vinylcaprolactam) and silica particles with 80 and 330 nm were synthesized to be employed as delivery systems for hydrophobic (naringin) and hydrophilic (doxorubicin hydrochloride) drugs. The insertion of SiO_2_ increased the rheological properties of the nanocomposite at 37 °C, which helps to prevent its diffusion away from the site of injection. The synthesized materials were also able to control the drug release for a period of 7 days under physiological conditions. Due to its higher hydrophobicity and better interaction with the PNVCL matrix, naringin presented a more controlled release. The Korsmeyer–Peppas model indicated different release mechanisms for each drug. At last, a preliminary *in vitro* study of DOX-loaded nanocomposites cultured with L929 and MB49 cells showed negligible toxic effects on healthy cells and better efficient inhibition of carcinoma cells.

## Introduction

Polymers are widely used for drug delivery systems due to their properties that allow the formulation of systems with high control over the release of different drugs for prolonged periods of time.^1^ The use of controlled release systems allows the concentration of the drug in the body to remain in the therapeutic range during the entire treatment period. Among the polymers used, the so-called smart-polymers stand out since they change their properties as a response to physical or chemical external stimuli.^2^ Thermoresponsive polymers present the advantage of being able to be applied as injectable systems, since they can be soluble and fluid at room temperature and become a gel in response to physiological temperature.^3,4^ Therefore, they do not require surgical procedures to be inserted *in vivo* and are able to be delivered through a syringe directly in tumors tissues, for example. This reduces surgical trauma and can easily mold in the free volume available, adapting to the surrounding tissue.^5−8^

Some pharmaceuticals, amino acids, and proteins have their treatment effectiveness reduced due to rapid degradation or inactivation of the compound after administration.^9^ Thermosensitive polymers have become a potential alternative to circumvent this limitation since they consist of a physical network of hydrophilic and hydrophobic groups that are able to interact with both the physiological medium and the active compound.^10,11^ Moreover, their physicochemical properties similar to those of living tissues, such as high-water content and elastic consistency, allow a prolonged protection of the drug while encapsulated, increasing their effectiveness, in addition to their localized release that can minimize side effects typically caused by systemic administration.^4,12−14^

Another common problem is the controlled release of hydrophobic drugs due to their low solubility, which results in low bioavailability when applied orally.^15^ The use of a thermosensitive polymer as a delivery system can avoid the need for the drug modification for its application. Many drugs are discarded even though they are highly effective in the treatment of diseases because they are not well absorbed by the human tissues.^16^ Thus, after application in the body through an injection at the desired site, the polymer would be able to encapsulate the hydrophobic drug due its temperature-induced phase transition and control its release.

Poly(*N*-vinylcaprolactam) (PNVCL) is a thermoresponsive polymer with increasing interest in the biomedical field due to its biocompatibility, non-toxicity, and lower critical solution temperature (LCST) close to physiological temperature.^17,18^ While PNVCL has a sharp temperature-induced phase transition, we recently demonstrated that the inclusion of silica nanoparticles in nanocomposite hydrogels resulted in a diffuse transition. It was attributed to the formation of intermediate globule states and a hydration/dehydration process that evolves in a wider temperature range.^19,20^ The presence of silica nanoparticles introduced a distinct profile for the temperature-induced phase transition, which could have great potential as a controlled drug delivery system.

**Figure 1:**
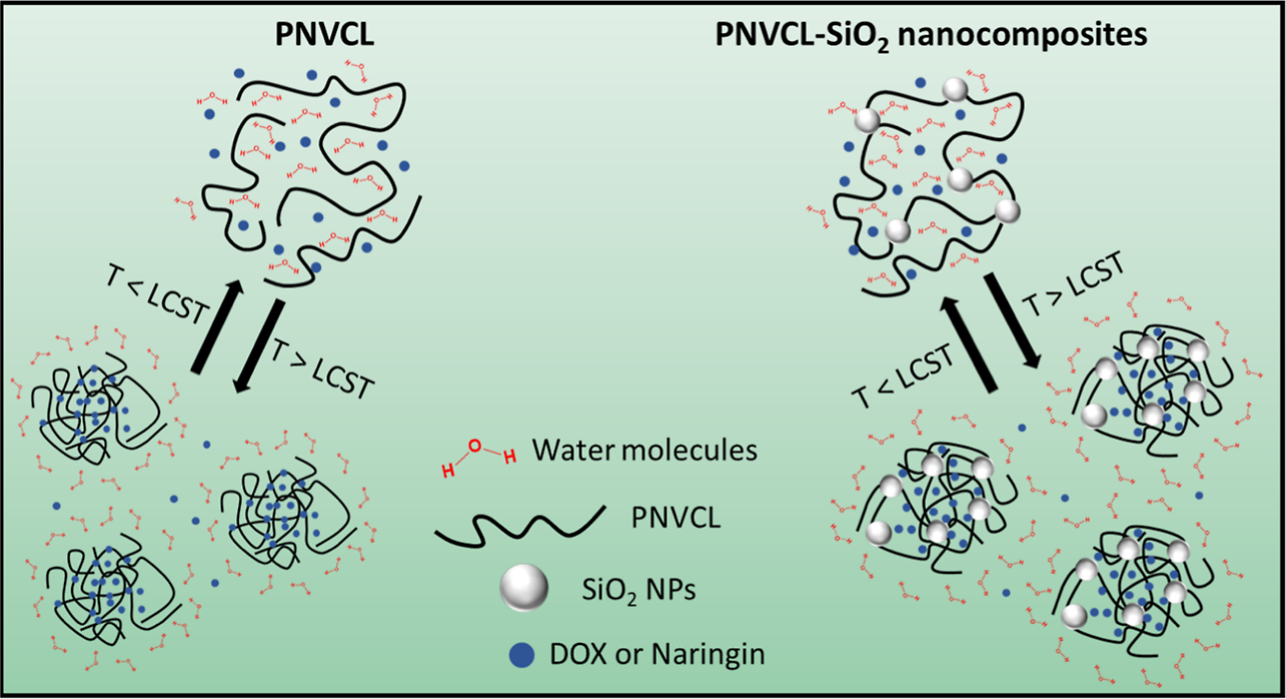
Controlled release systems used in these studies are based on PNVCL and silica nanospheres. At room temperature, both the pure polymer and the nanocomposites with 5 wt % SiO_2_ are soluble in the drugs such as doxorubicin and naringin. However, when heated above the LCST, PNVCL chains aggregate, expelling water molecules and trapping the drug molecules in the structure. This results in the separation of phases, causing the polymer to become more viscous and controlling the localized release of drugs. It is worth noting that the transition is reversible, meaning that the system can be made soluble again by cooling to a temperature below the LCST.

Therefore, in this work, we present a methodology for obtaining thermosensitive injectable nanocomposites of PNVCL and silica nanoparticles with spherical morphology. These materials were obtained *in situ*, and the nanoparticles were covalently bonded to the polymeric matrix. The effect of temperature and the addition of nanoparticles on the rheological properties of the polymers was evaluated. Moreover, these materials were used as delivery systems for the release of hydrophobic (naringin) and hydrophilic (doxorubicin hydrochloride) drugs at the physiological temperature and pH (Figure 1). With the application of kinetic models on the cumulative release curves, it was possible to determine the mechanism of release from the PNVCL matrix for both drugs. Based on the results, a preliminary DOX release test was performed on healthy cells (L929) and on bladder carcinoma cells (MB49). It is proposed that PNVCL-SiO_2_ thermosensitive nanocomposites could be used as drug carriers in future pharmaceutical applications.

## Materials and Methods

### Synthesis of SiO_2_ Spheres

The SiO_2_ spherical nanoparticles were synthesized using the methodology previously described by our group.^20,21^ Spherical nanoparticles of two different sizes (80 and 330 nm) were obtained by varying the amount of ammonium hydroxide (NH_4_OH 30%) (1 and 2 mL). These nanoparticles were functionalized with 3-methacryloxypropyltrimethoxysilane (MPS) to allow the spheres to covalently bond with the PNVCL chains during polymerization. Thus, 0.842 mmol of 3-(trimethoxysilyl)propyl methacrylate (MPS) (Alpha Aesar 98%) was added after 4 h of reaction, and the mixture was stirred at room temperature for another 20 h. Subsequently, the nanoparticles were purified by three consecutive steps of centrifugation at 8500 rpm for 6 min and washing with anhydrous ethanol.

### Synthesis of PNVCL and Nanocomposites

PNVCL and its nanocomposites with 5 wt % of silica nanoparticles were synthesized based on the radical polymerization procedure previous described by our group.^19,20,22,23^ For this synthesis, 0.0360 mol of monomer *N*-vinylcaprolactam (NVCL) dissolved in 18 mL of anhydrous dimethyl sulfoxide (DMSO) was added to the reactor together with the nanoparticles, in the case of the nanocomposites. Then, 0.682 mmol of the azobisisobutyronitrile (AIBN) initiator dissolved in 7.6 mL of DMSO was added slowly onto this solution. The reaction proceeded at 70 °C, during 4 h, under a nitrogen atmosphere, and the obtained polymers were purified by dialysis against distilled water for 3 days using a membrane tube with a M_w_ cutoff of 3500 Da. The amount of silica used was 5% in relation to the initial mass of the monomer. The materials were labeled NC-80 and NC-300 for nanocomposites synthesized with nanoparticles with diameters of 80 and 330 nm, respectively.

### Characterization

The infrared spectra of SiO_2_ nanoparticles in the range 400–4000 cm^–1^ were collected by diffuse reflectance infrared Fourier transform (DRIFT) spectroscopy (Bruker Equinox 55) to confirm the functionalization. Scanning electron microscopy (SEM-FEG, ZEISS model-SUPRA 35) was used to determine the morphology and the size distribution of the silica nanoparticles.

To assess the effect of the silica nanospheres on the polymerization process, ^1^H NMR spectra of PNVCL and its nanocomposites were collected by liquid-state NMR experiments on a Bruker AVANCE III spectrometer operating in a magnetic field of 9.4 T Oxford, with a related frequency of 400 MHz for the hydrogen-1 nucleus. PNVCL and nanocomposite samples were solubilized in water, using deuterium as an external standard. All experiments were performed at room temperature.

The cloud point temperature (*T*_cp_) of the synthesized polymers was determined by the change in transmittance of aqueous solutions with a concentration of 1 % wt. The *T*_cp_ value can be calculated as the temperature at which the transmittance of the system is equal to half of the initial transmittance^30^. The spectra were collected in a Multi-Spec-1501 UV–vis spectrophotometer Shimadzu with a TCC-240A thermoelectrically temperature-controlled cell holder. The wavelength range of 200–800 nm and the temperature interval of 25.0–35.5 °C were used. Spectra were collected every 0.5 °C with an interval of 3 min between each measurement.

Rheological analysis was performed to evaluate the influence of silica nanospheres on the viscoelastic properties of polymers at 25 and 37 °C. Solutions with a concentration of 20 wt % PNVCL, NC-80, and NC-330 were analyzed in an Anton-Paar (Modular Compact Rheometer, Graz, Austria) model MCR 302, equipped with a parallel geometry plate with 25 mm diameter and a gap of 0.5 mm. The time sweep analysis was performed using a strain of 5.0% and frequency of 1 Hz during a 10 min interval.

### *In Vitro* Release of Naringin and Doxorubicin (DOX)

Naringin and doxorubicin hydrochloride (DOX) were used to assess the ability of PNVCL to absorb and release molecules in a controlled manner. The experiment was performed in a thermostatic bath (Nova Ética, Brazil) at a temperature of 37 °C and pH 7.4. Each material (200 mg) was added in 1 mL of a 100 ppm solution of DOX or in 100 ppm solution of naringin in PBS and kept at 5 °C for 24 h for total solubilization. Then, 300 μL of this solution was added to a vial and incubated for 15 min in the thermostatic bath for the transition, followed by the addition of 1.8 mL of PBS. At predetermined times, 1 mL of medium was taken and replaced by fresh solution buffer. The DOX aliquots were analyzed in a UV–vis spectrometer at 480 nm and 280 nm for naringin.

### Erosion Test

The erosion test was carried out in a thermostatic bath at a temperature of 37 °C. The polymeric solution was prepared by adding 60 mg of pure PNVCL in a small vial together with 300 μL of PBS (20 wt %). This mixture was maintained at 5 °C for 24 h for total solubilization. Then, the solution was placed in the thermostatic bath for 15 min for the transition with the addition of 1.8 mL of PBS. After 7 days, the media was removed and the remaining polymers were dried in an oven at 70 °C for 3 days to determine the residual mass of PNVCL and its nanocomposites.

To evaluate the polymer molar mass variation before and after the erosion tests, the size exclusion chromatography (SEC) technique was used. Analysis of 200 μL of PNVCL solution with a concentration of 5 wt % was performed in a Viscotek HT-GPC (Malvern) with three H-806 M columns (mixed) and a refractive index detector, employing tetrahydrofuran (THF) as an eluent at 50 °C and a flow rate of 1 mL/min. Calibration was carried out using narrowly distributed standards of polystyrene (500 to 2,500,000 g/mol).

### Cell Culture

Murine bladder tumor cells (MB49) and fibroblasts (L929) were maintained in an incubator at 37 °C, 5% CO_2_, and 95% humidity and in Dulbecco’s modified Eagle’s medium—DMEM (Gibco, Life Technologies, USA) with high glucose levels, supplemented with 10% fetal bovine serum (Gibco, Life Technologies, USA), 4.5 g/L glucose, 2 mM l-glutamine, 1.5 g/L NaHCO_3_, 1% penicillin antibiotic (100 U/L mL), and streptomycin (100 μg/mL) (complete culture medium). Cell passages were performed by trypsinization every 2 days. Cells were maintained in culture until reaching 90% confluence.

### Cell Viability on Exposure to PNVCL-DOX

Cell viability was assessed using an indirect method based on the work of Pereira et al.,^24^ who also tested a composite hydrogel. Cell lines MB49 and L929 were plated in 96-well culture plates (Corning Incorporated, NY, USA) at a concentration of 1 × 10^5^ cells/well in complete DMEM and maintained for the period of 24 h for cell adherence in an incubator at 37 °C, 5% CO_2_, and 95% humidity. To evaluate the effect of DOX release by the polymers, 300 μL of samples of 20 wt % hydrogels (in PBS) containing 0.345 μM DOX was incubated in a water bath at 37 °C for 15 min with subsequent addition of 1.8 mL of complete DMEM culture medium and released for 24 h. Then, 100 μL of the supernatant released media was added to the cells. Unlike the previous test in which the release medium was PBS, in this case, the test was performed in a culture medium to allow the addition of the aliquot of drugs released into the cells. We did not perform a test using only free DOX, since this methodology would not mimic the conditions of controlled release and the entire dosage of the drug would be applied at once to the cells. After 24 h, the cells were washed carefully with PBS buffer (1×), and the cell viability test was performed by adding 200 μL/well of a 70 mM Resazurin solution (Sigma-Aldrich, USA). The cells were kept for 4 h in this solution, and the absorbance readings were taken on a spectrophotometer between wavelengths of 570 and 600 nm.

## Results and Discussion

The spherical nanoparticles were synthesized by a procedure based on the Stöber method.^25^ These nanoparticles were described in a previous work by our group in which we studied their effect on the sol–gel transition of PNVCL hydrogels.^19,20^Figure S1 showed nanoparticles with spherical morphology and average sizes of 80 ± 13 nm (when 1 mL of NH_4_OH was added to the synthesis) and 330 ± 21 nm (2 mL of NH_4_OH). The surface of the nanoparticles was functionalized with the organosilane agent MPS.^26^ The functionalization process allows the nanoparticles to bind covalently to the PNVCL chains during its polymerization.^19,20^ As shown in Figure S2, the functionalization was confirmed by the presence of CH_2_ and C=O groups in the infrared spectra that did not appear in the spectrum of bare silica.^27,28^

**Figure 2:**
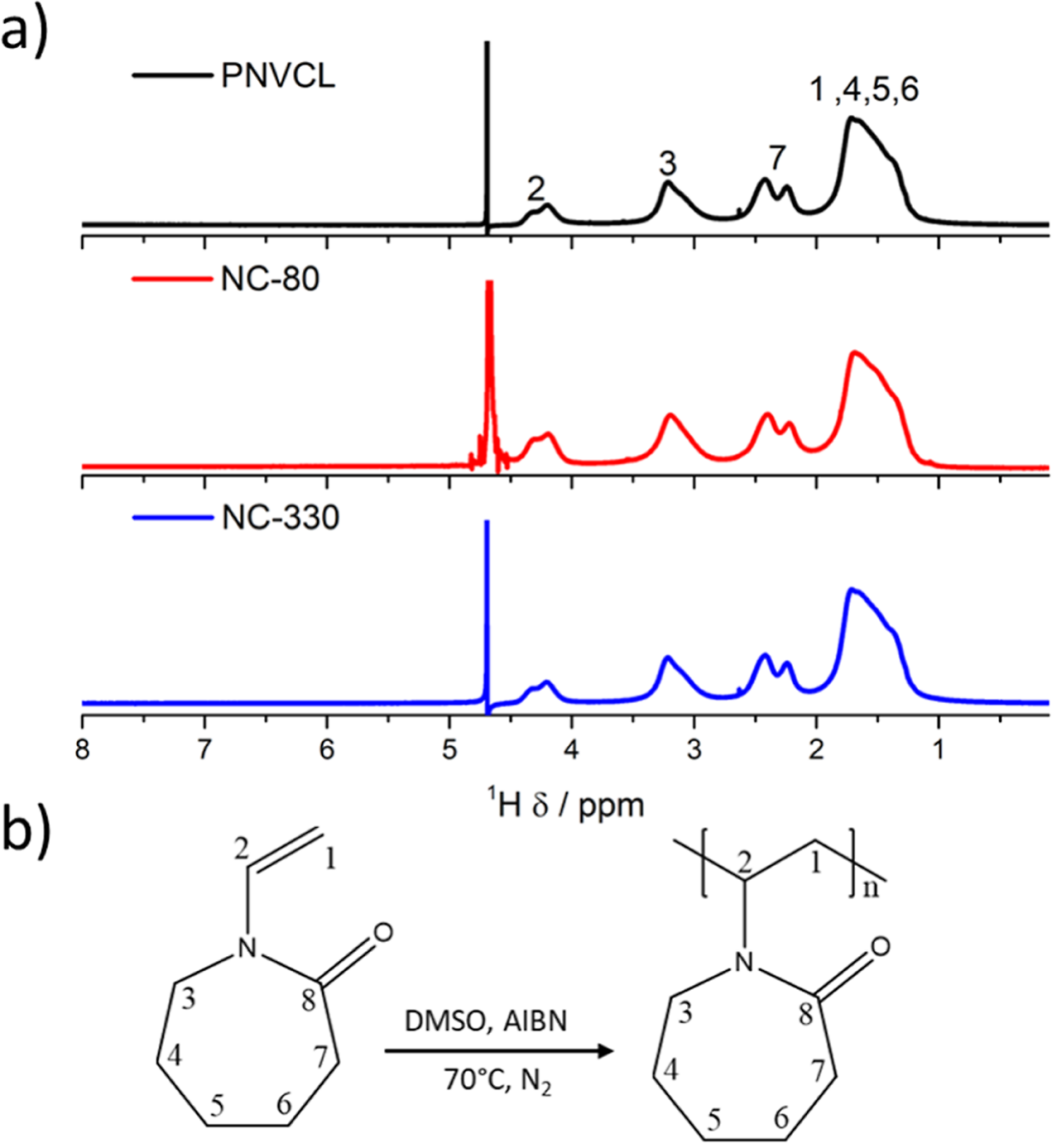
^1^H NMR spectra of pure PNVCL and its nanocomposites NC-80 and NC-330 with 5% of silica (a) and scheme showing the polymerization process of PNVCL (b).

The nanocomposites were prepared *in situ* with the addition of 5 wt % of silica nanoparticles to the monomer amount used for the polymerization. We have previously demonstrated that the insertion of the nanoparticles did not affect the polymerization process since the ^1^H NMR spectra of the nanocomposites and the pure polymer do not differ.^19,20^Figure 2a shows the spectra of the PNVCL and its nanocomposites and the corresponding protons from the vinyl group and the −CH_2_ in the ring (δ 1.20–1.91 ppm), −CH_2_ adjacent to the C=O group (δ 2.10–2.60 ppm), −CH_2_ of the ring adjacent to the nitrogen atom (δ 2.91–336 ppm), and −CH attached to the nitrogen atom (δ 4.01–4.44 ppm). The purification of the nanocomposites and PNVCL by dialysis proved to be effective for removing unreacted monomers since there is no residual proton around δ 7 ppm related to the proton from the carbon 1 of NVCL, as shown in Figure 1b.

**Figure 3:**
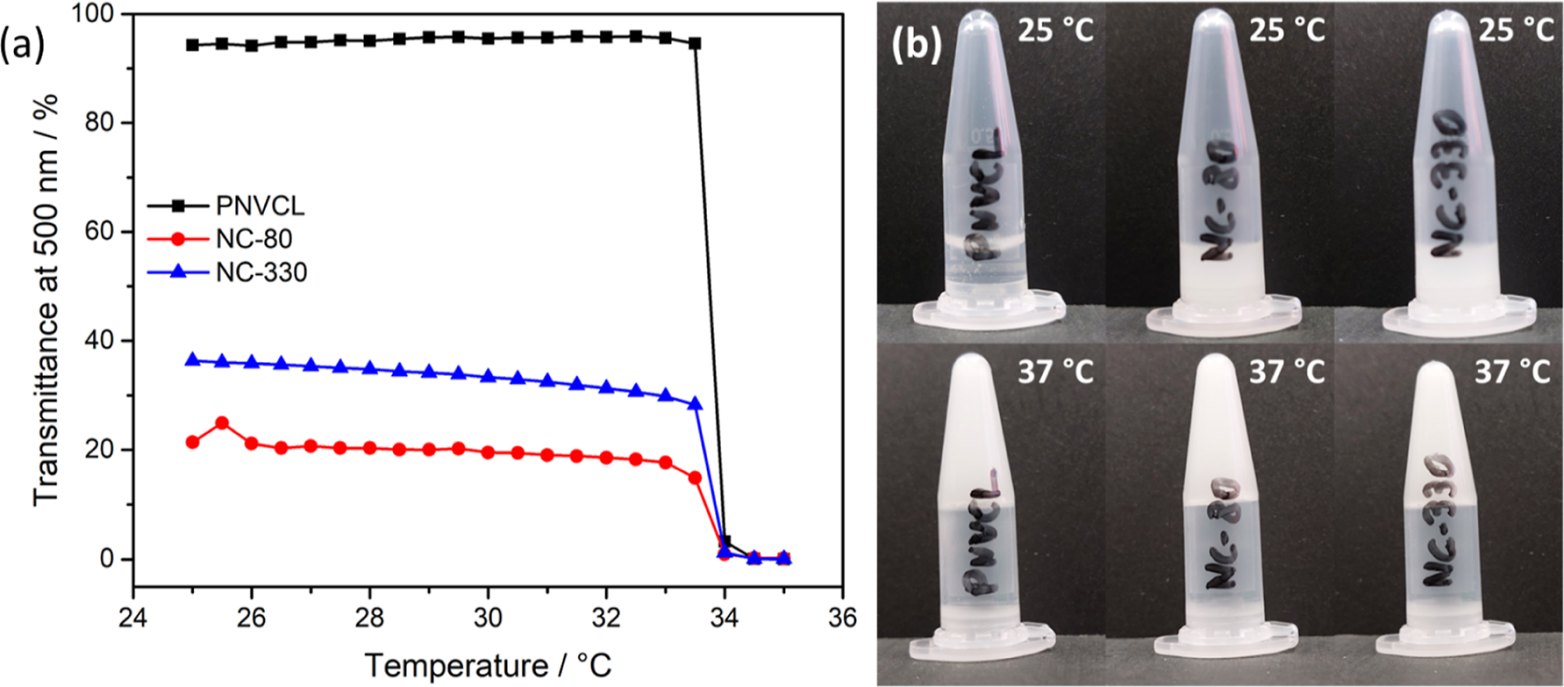
(a) Transmittance variation at 500 nm of 1 wt % solutions of PNVCL and its nanocomposites with increasing temperature and (b) images of inverted vials containing 20 wt % PNVCL and nanocomposite solutions at room temperature and after being heated to 37 °C.

When thermosensitive polymers dispersed in aqueous solution are heated above a critical temperature, they change from a solvated to an agglomerated state. This occurs as the entropy of the mixture decreases and the hydrogen bonds between the polymer and the water molecules break. As a consequence, the polymer chains start to agglomerate by dipole–dipole interactions and van der Waals forces and expel the water molecules, resulting in a phase separation.^29^ The cloud point temperature (*T*_cp_) was determined by evaluating the change in transmittance with temperature of 1 wt % aqueous solution of the pure polymer and nanocomposites. Figure 3a shows the transmittance variation at an intermediate wavelength (500 nm) in the temperature range of 25 to 35 °C for all materials. The nanocomposites presented a lower transmittance due to the presence of the opaque silica nanoparticles. All materials showed *T*_cp_ close to 34 °C; however, the transmittance of the nanocomposites gradually decreased before the transition. In previous studies, we showed that this effect occurs because the nanoparticles affect the interactions between the polymer and the water molecules, facilitating the agglomeration.^19,20^Figure 3b indicates that all the materials at a concentration of 20 wt % can be applied as injectable systems for drug delivery, as they gel at physiological temperature (37 °C), becoming a viscoelastic material that does not flow when inverted. A rapid release of the loaded drug can occur when a polymer fraction remains soluble after application. To avoid this problem, it is recommended that the polymer has the ability to gel quickly at a temperature below and close to 37 °C.^31^

In order to evaluate the effect on the rheological properties of PNVCL and its injectability after adding silica nanospheres, the storage (*G*′) and loss (*G*″) modules and the complex viscosity (η*) were analyzed at temperatures of 25 and 37 °C. All tests were performed in the linear viscoelastic region determined by an amplitude sweep test (Figure S4). A concentration of 20 wt % was chosen for the rheological tests based on the work of Sala et al. on the application of PNVCL for cartilage tissue engineering. This concentration permitted the*in vivo* administration of the polymer using a syringe, in addition to allowing the material to remain stable at the application site after the transition.^23^

**Figure 4:**
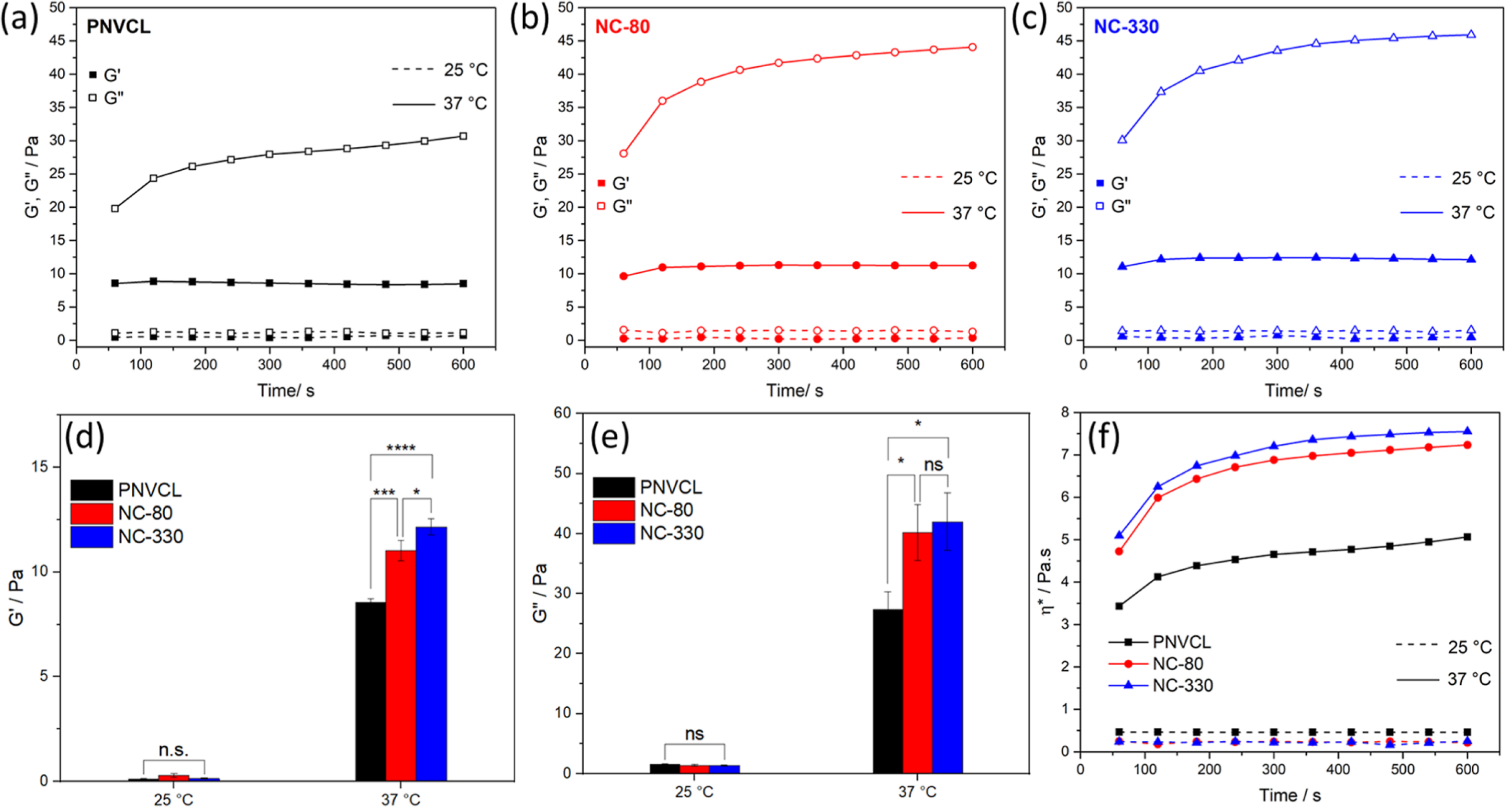
Storage (*G*′) and loss (*G*″) moduli of 20 wt % solutions of PNVCL (a), NC-80 (b), and NC-330 (c) at 25 and 37 °C. Comparison of *G*′ (d) and *G*″ (e) obtained in time sweep tests using a frequency of 1 Hz and 5% strain and at different temperatures. The complex viscosity of the thermoresponsive PNVCL and its nanocomposites increases upon heating to the physiological temperature (f). Statistical analysis was performed using Graph Pad Prism 9 software and one way ANOVA test. Statistical significance was assumed for *p*-values < 0.05: *P < 0.05, **P < 0.01, ***P < 0.001, and ****P < 0.0001.

Figure 4a shows that after increasing the temperature from 25 to 37 °C, the PNVCL solution presented an increase in both loss and storage moduli. This increase is related to the transition of the material from a solvated to an agglomerated state when heated above its LCST. The transition of the thermosensitive polymer can be accompanied by the formation of a hydrogel and the crossover of the modules (*G*′ > *G*″), as the PNVCL chains form a structure with reversible cross-linking. However, even after heating at the physiological temperature, the values of *G*″ remained higher than the values of *G*′, indicating an increase in the rheological properties of the material without the formation of a hydrogel. Halligan et al. also reported *G*″ > *G*′ for 3 wt % solutions of PNVCL at 37 °C with the storage modulus surpassing the loss modulus only at 47 °C, showing that our system could result in a hydrogel if heated at higher temperatures.^32^

The same results were observed for the nanocomposites at both temperatures, as shown in Figure 4b,c. Even though a hydrogel was not obtained, the presence of silica nanoparticles in the polymeric matrix resulted in higher values of *G*′ and *G*″ after the transition at 37 °C. Figure 4d,e shows the modulus curves of all polymers before and after the transition. The addition of the nanospheres in the percentage of 5% increased *G*′ and *G*″ in 35 and 50%, respectively, in comparison to the pure polymer. This improvement can be explained by the creation of an interconnected network of polymeric chains and silica, which contributed to the increase in the elastic portion of the nanocomposite.^33^

By analyzing Figure 4f, it is possible to see that there is no difference in the values of complex viscosity after the addition of the nanoparticles at room temperature. The low values of viscosity indicate that these solutions at a concentration of 20 wt % can be used as injectable systems, since the materials used for injectable applications need to present a low viscosity to allow their administration by syringe.^34^ When the temperature of the polymer solutions is increased to 37 °C, the temperature-induced phase transition is confirmed by a pronounced increase in η*. At the physiological temperature, the nanocomposites showed higher complex viscosity values than the pure polymer, indicating that the silica was able to increase the viscoelastic properties of PNVCL. This increase can be advantageous, as low viscosity materials can result in premature dissolution of the system, leading to rapid drug release and eventual leakage of the material from the target site.^10^ We previously demonstrated that^19,20^ when functionalized silica nanoparticles are incorporated during the polymerization of PNVCL, they act as a cross-linking agent in the gel state, which could also explain the increase in η* above the phase transition.

After confirming the ability of the PNVCL and nanocomposites to undergo the sol–gel transition at physiological temperature with gain of rheological properties, the release of two drugs with different hydrophilicities was tested. Naringin (flavanone-7-O-glycoside) is a hydrophobic flavonoid that stands out among the molecules used to treat bone diseases. Due to its pro-osteogenic effect, it can be used in the treatment of osteoporosis as a mediator in the osteogenic differentiation of mesenchymal stem cells.^35^ Another drug of great importance is doxorubicin (DOX) which is widely used as a chemotherapy drug to treat cancers in the ovary, breast, lung, and bladder.^36^ The compound in the hydrochloride form is hydrophilic and therefore can serve as a comparison with naringin.

**Figure 5:**
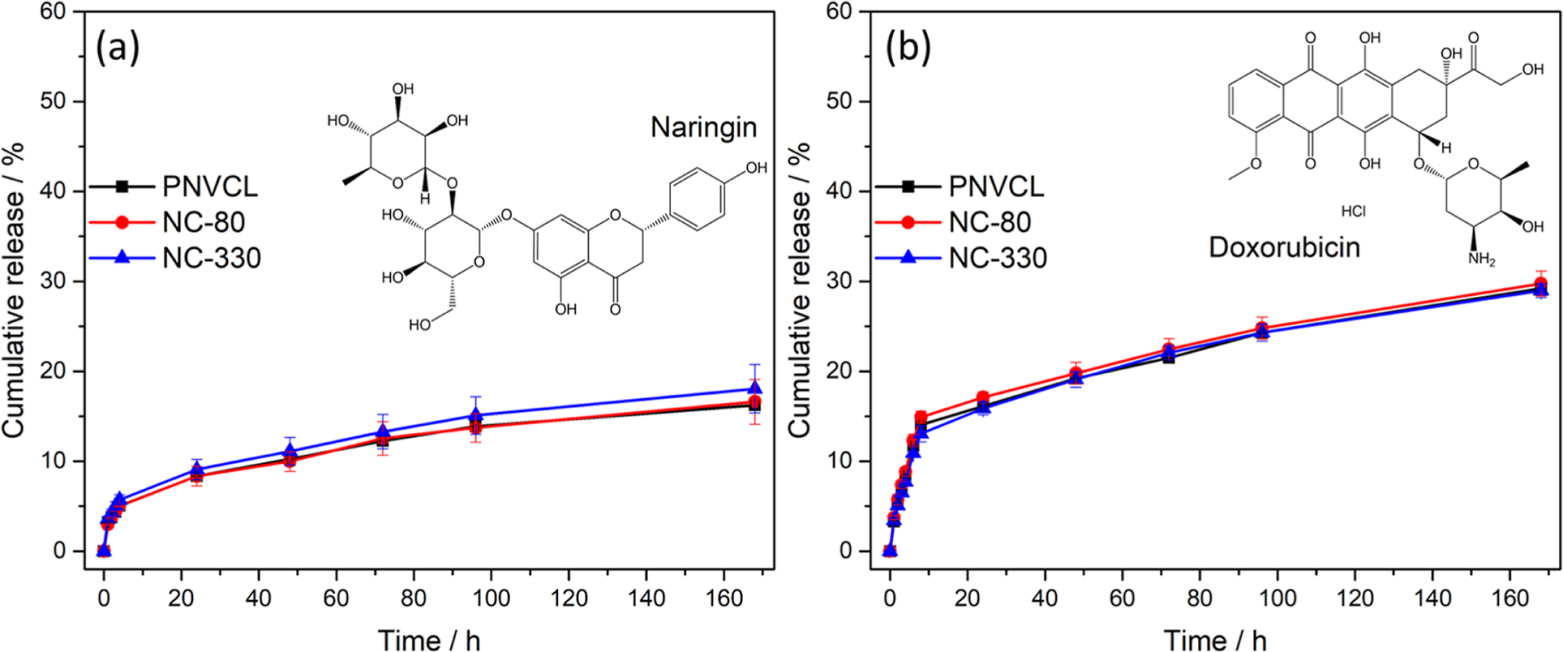
Release profile curves of naringin (a) and doxorubicin hydrochloride (b) at 37 °C and pH 7.4. PNVCL and nanocomposite solutions (20 wt %) were heated for 15 min before the addition of PBS to ensure the complete transition to the agglomerated state.

Figure 5a,b shows, respectively, the naringin and DOX release profiles at pH 7.4 and 37 °C of PNVCL and nanocomposites (20 wt %) loaded with 100 ppm of drugs. After 7 days of test, the polymers released 17% of total naringin encapsulated and 30% of DOX. This difference of release can be explained by the better interaction between the PNVCL matrix and naringin. The polymer chains begin to agglomerate when heated to physiological temperature and predominantly form hydrophobic interactions. In this way, the drug experiences a hydrophobic environment within the polymer, which allows a better control over the release of naringin. On the other hand, since DOX is a hydrophilic molecule, its cumulative release is greater in the same time interval, being almost twice faster than naringin. Furthermore, the insertion of nanoparticles did not affect the release of both drugs. This result shows that despite the increase in the viscosity and overall changes in the polymer structure and thermoresponsive profile by the insertion of silica nanoparticles, the release was the same using PNVCL and its nanocomposites.

To determine the release mechanism of naringin and DOX, the zero-order, first-order, Higuchi, and Korsmeyer–Peppas models were applied to the curves shown in Figure 5. The parameters calculated are presented in Table 1. The model that best fits the curves was Korsmeyer–Peppas, presenting the highest *R*^2^ values under all conditions. In the exponential Korsmeyer–Peppas model, the exponent is variable and is represented by *n*, which indicates the type of release mechanism. When *n* ≤ 0.50, the release is controlled by diffusion, whereas values between 0.50 and 1.00 mean anomalous transport and the release is controlled both by the diffusion effect and by the erosion of the polymeric matrix.^37^ The calculated *n* values for naringin were below 0.5, indicating that the mechanism that controls the flavonoid release is its diffusion into the supernatant, independent of polymer erosion. However, for doxorubicin, the values found were in the range 0.5 < *n* < 1.0, indicating an anomalous transport mechanism. This means that the release of DOX depends on both the diffusion of the molecule to the supernatant and the erosion of the PNVCL. These results corroborate with the greater interaction between the polymer and naringin, since this strong interaction hinders the release of the flavonoid and its diffusion out of the polymeric matrix becomes the determining step of the release.

**Table 1:** Parameters Obtained after Applying the Kinetic Models to Naringin and DOX Release Curves Shown in Figure 5

		naringin			DOX		
material	kinetic model	*K*	*n*	*R*^2^	*K*	*n*	*R*^2^
PNVCL	zero-order	0.00189		0.778	0.00227		0.753
	first-order	0.00213		0.804	0.00261		0.786
	Higuchi	0.888		0.952	0.0886		0.954
	Korsmeyer–Peppas	0.228	0.333	0.969	0.111	0.703	0.998
NC-80	zero-order	0.00179		0.766	0.00233		0.739
	first-order	0.00201		0.791	0.00269		0.773
	Higuchi	0.087		0.944	0.0898		0.947
	Korsmeyer–Peppas	0.232	0.376	0.992	0.122	0.669	0.996
NC-330	zero-order	0.00176		0.905	0.00226		0.766
	first-order	0.00196		0.913	0.00261		0.799
	Higuchi	0.908		0.918	0.0888		0.962
	Korsmeyer–Peppas	0.279	0.335	0.968	0.114	0.650	0.993

Chang et al.^38^ studied naringin release using a thermosensitive polymer made of amphipathic carboxymethyl-hexanoyl chitosan (CHC), β-glycerol phosphate (β-GP), and glycerol. At physiological pH, the release was above 40% after the first 24 h and almost 100% after 5 days of testing. When compared to our study, it is clear that the use of PNVCL promotes greater control over naringin release, reaching 17% after 1 week, and therefore allows the creation of a system for treatments that require a longer duration. Zhang et al.^4^ studied the release of DOX from an injectable thermosensitive polymer, using a polymeric matrix based on chitosan and hyaluronic acid. Although the release values after 1 week are similar to ours, the values of *n* found were below 0.50, meaning that the release of DOX from the chitosan matrix is controlled only by diffusion. On the other hand, the PNVCL matrix allows the DOX release to be controlled by modification in the interactions between the polymeric chains, which control hydrogel erosion.

In order to evaluate the stability of polymers under physiological conditions, an erosion test was carried out under the same conditions used for the release test but without the drugs. The final polymer mass after the test was determined and compared to the initial mass. The pure polymer lost 4.7 ± 0.4% of its initial mass, while the NC-80 and NC-330 nanocomposites lost, respectively, 7.2 ± 0.2 and 6.0 ± 0.1% after 7 days. Despite being a small mass loss, the test showed that the PNVCL undergoes an erosion process under physiological conditions.

Although the polymer is undergoing a mass loss process, it is not yet possible to determine whether the mechanism of this event is through dissolution of the polymer to the supernatant or whether the polymer chains are degrading. To determine the erosion mechanism, an SEC analysis was performed on the samples of pure PNVCL after the erosion test. As can be seen in Figure S3, there is no difference in retention time between a freshly prepared aqueous PNVCL solution and its supernatant solutions after keeping PNVCL hydrogels at pH 7.4 for 7 days. Since there is no difference between the average molar mass of PNVCL samples before and after the erosion test, it is possible to infer that the dissolution of PNVCL and its nanocomposites is the main erosion mechanism for drug release.

Considering that the polymers were able to control the release of DOX, a preliminary test of the release of the anticancer drug in two different cells was performed: murine bladder tumor cells (MB49) and mouse fibroblasts (L929).^39^ The polymers without drugs were also tested, allowing to evaluate the viability of these cells against the polymers with and without DOX. The viability test was performed by the indirect method, in which the supernatant containing the content released by the polymers after a period of 24 h was added to the cells, which were then cultured for 24 h in this solution.

**Figure 6:**
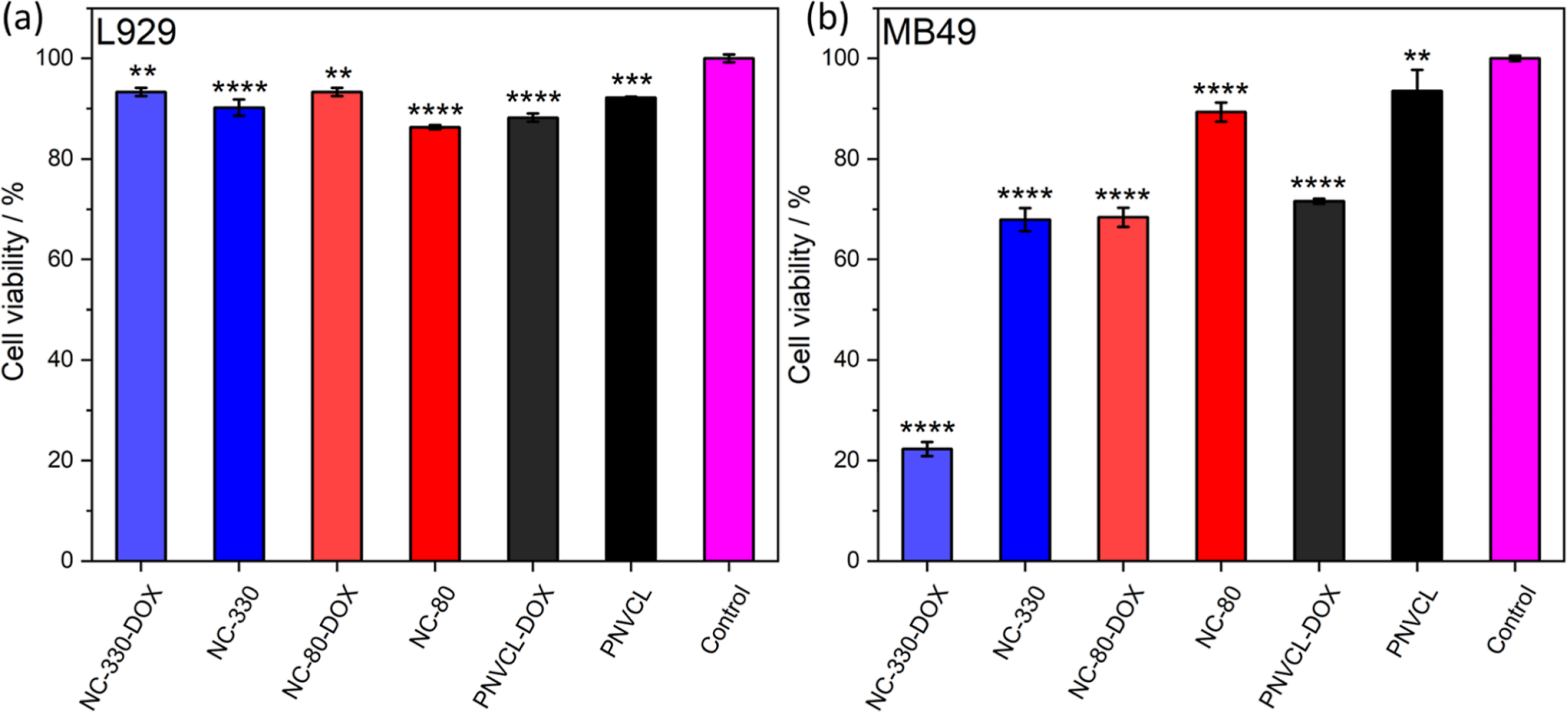
Indirect cell viability determination performed on L929 (a) and MB49 (b) cells (1 × 10^5^ cells/well) treated with extracts of DOX delivery test of 24 h. Polymeric solutions (20 wt %) were incubated without or with DOX (0.345 μM) for 15 min at 37 °C for complete transition to the agglomerated state with subsequent addition of DMEM. After 24 h, 100 μL of the supernatant was added to the cells to assess the effect of both the polymer and drug on cell viability. Statistical analysis was performed using Graph Pad Prism 9 software and one way ANOVA test. Statistical significance was assumed for *p*-values < 0.05: *P < 0.05, **P < 0.01, ***P < 0.001, and ****P < 0.0001.

Figure 6a shows the viability of L929 cells after 24 h of culture in the released solution from the polymers. As previously shown, the polymers undergo an erosion process under physiological conditions, releasing polymeric chains in the case of pure PNVCL and chains with silica nanoparticles in the case of nanocomposites. This was confirmed by dynamic light scattering (DLS), since PNVCL presented a single size distribution curve and the nanocomposite presented two distributions curves, related to the polymeric chains and the silica nanoparticles (Figure S6). When these materials are loaded with drugs, these molecules are also released out of the PNVCL matrix. Thus, the aliquots added to the cells presented different compositions depending on the formulation used. When the polymers without the drug dosage were used, it is possible to see that the viability remained close to 90% in relation to the control group. This result confirms that both pure PNVCL and its nanocomposites with silica nanospheres are biocompatible with healthy fibroblast cells. Similar results were also obtained for DOX-loaded polymers systems, showing that the dose of anticancer released in 24 h was not enough to cause toxicity to L929 cells. This observation is in agreement with the result previously reported by Lanks and Lehman, who showed that DOX is not able to kill L929 cells at therapeutic concentrations.^40^

The same methodology was applied to MB49 cancer cells, and the results after 24 h can be seen in Figure 6b. Similarly, PNVCL and NC-80 materials resulted in an cell viability close to 90%, showing that these polymers also do not cause toxicity to this cell line. However, the content released from the NC-330 nanocomposite resulted in a reduced viability (70%). Different from L929 cells, the addition of drugs to polymer systems decreased the viability of tumor cells to 70% for PNVCL and NC-80 and 20% for NC-330 after 24 h. This larger drop indicates a synergistic effect between NC-330 and doxorubicin, with the nanocomposite increasing the effectiveness of the anticancer drug. Considering that the only difference between NC-80 and NC-330 is the size of the silica nanospheres, it is possible that the greater toxicity is due to the larger size of the nanoparticles. Similar findings were reported for THP-1 and endothelial (EC) cells using amorphous silica nanoparticles of different sizes.^41,42^ When these cells were treated with nanoparticles that ranged in size from 16 to 1000 nm, silica particles smaller than 80 nm did not show any toxicity and had similar viability to the control. However, when using 300 nm nanoparticles, both cell lines’ metabolic activity decreased by 40% and there was an increase in lactate dehydrogenase (LDH) release, indicating cell death and membrane damage. Further experiments were conducted to understand the cell death mechanism caused by these larger nanoparticles. After 24 h, there was no increase in caspase activity in either THP-1 or EC cells, suggesting that necrotic mechanisms may be involved in the toxic effect of larger silica nanoparticles. These findings support the evidence of higher toxicity in the NC-330 nanocomposite. Furthermore, previous works on the controlled release of DOX on MB49 cells using drug delivery systems also reported the high toxicity of this drug on this type of cell.^43−45^ The results shown in Figure 6b confirm the toxicity of DOX on carcinoma cells since its presence resulted in lower viability when compared to formulations containing only thermosensitive polymers.

## Conclusions

In summary, we developed thermosensitive systems based on PNVCL and silica nanoparticles with temperature-induced phase transition close to 34 °C and ability to incorporate hydrophobic and hydrophilic pharmaceuticals for localized drug delivery. The rheological analysis of 20 wt % polymer solutions showed an increase in the viscoelastic properties when heated from 25 to 37 °C, demonstrating that they can be locally injected to target sites. The incorporation of 5% of silica nanoparticles led to an increase in the complex viscosity and storage and loss moduli, which could minimize premature material dissolution and rapid drug release from the target site.

The synthesized polymers showed great capacity to sustain a controlled release of naringin and doxorubicin under physiological conditions for 7 days. The greater DOX release compared to naringin can be explained by the higher hydrophobic character of the latter, which interacted better with PNVCL and resulted in a higher control of its release. Kinetic analysis showed that the release of naringin is controlled by diffusion, while the release of DOX is controlled by anomalous transport. These results show that it is possible to use injectable PNVCL-based thermosensitive nanocomposites with silica nanospheres for both hydrophilic and hydrophobic drug delivery with high control and in a minimally invasive manner. In addition, a preliminary test showed that a possible treatment for bladder cancer employing PNVCL modified with silica nanospheres would control DOX release and potentially minimize side effects due to its low effect on healthy cells.

## Supporting Information Available

SEM images of the silica nanoparticles, infrared spectra of the silica nanoparticles, retention time of the PNVCL after the erosion test in different pH values, amplitude sweep test of PNVCL, and analysis of the hydrodynamic diameter for PNVCL and NC-330 measured in the DLS after the erosion tests (PDF)
