# Supplementary material for: Injectable Thermosensitive Nanocomposites Based on Poly(N-vinylcaprolactam) and Silica Particles for Localized Release of Hydrophilic and Hydrophobic Drugs
Source: Langmuir. 2023 Feb 6;39(6):2380–8. doi: 10.1021/acs.langmuir.2c03160 (PMC9933531; doi:10.1021/acs.langmuir.2c03160)
Supplement: Supplementary file 1 — la2c03160_si_001.pdf [file la2c03160_si_001.pdf]

Supporting Information

Injectable thermosensitive nanocomposites based on poly(N-vinylcaprolactam) and silica particles for localized release of hydrophilic and hydrophobic drugs

Lucas S. Ribeiro [a], Renata L. Sala [a,†], Thaiane A. Robeldo [b], Ricardo C. Borra [b], Emerson R. Camargo [a*]*

[a] Interdisciplinary Laboratory of Electrochemistry and Ceramics (LIEC), Departament of Chemistry, Federal University of São Carlos (UFSCar), Rod. Washington Luis km 235, CP 676 São Carlos, SP 13565-905, Brazil.

[b] Laboratory of Applied Immunology, Federal University of São Carlos (UFSCar), São Carlos, Rod. Washington Luis km 235, CP 676 São Carlos, SP 13565-905, Brazil.

[†] Present address: Department of Chemistry, University of Cambridge, Lensfield Road, Cambridge CB2 1EW, UK.

* Corresponding authors: rl635@cam.ac.uk and camargo@ufscar.br

Table of contents:

- SEM images of the silica nanoparticles (Figure S1 and Figure S2).
- Infrared spectra of the silica nanoparticles (Figure S3).
- (GPC) chromatograms of PNVCL before and after the erosion test (Figure S4).
- Amplitude sweep test of PNVCL (Figure S5).

- Analysis of the hydrodynamic diameter for PNVCL and NC-330 measured in the DLS after the erosion tests.

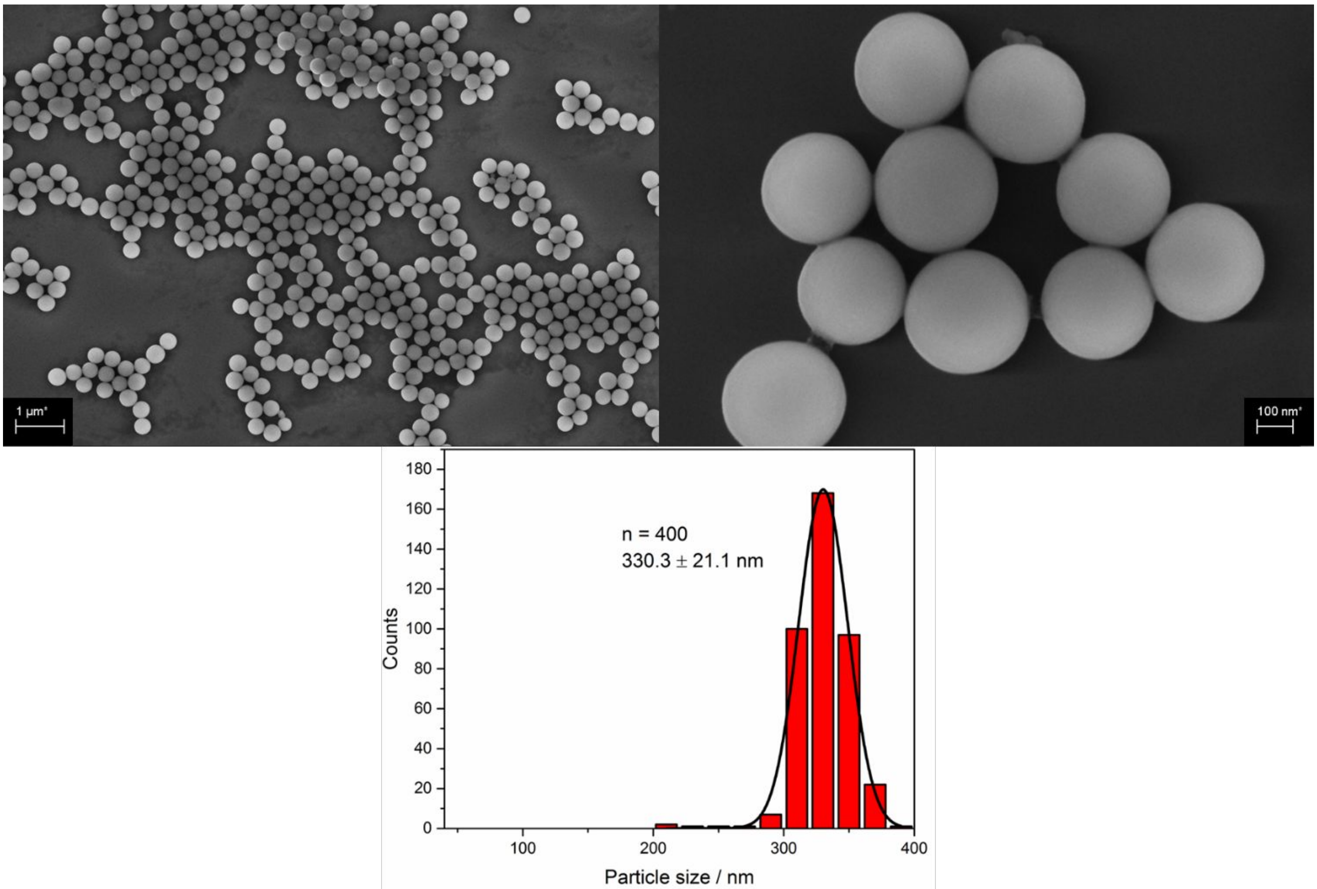

Fig. S1. SEM image of the silica nanoparticles NP-330.

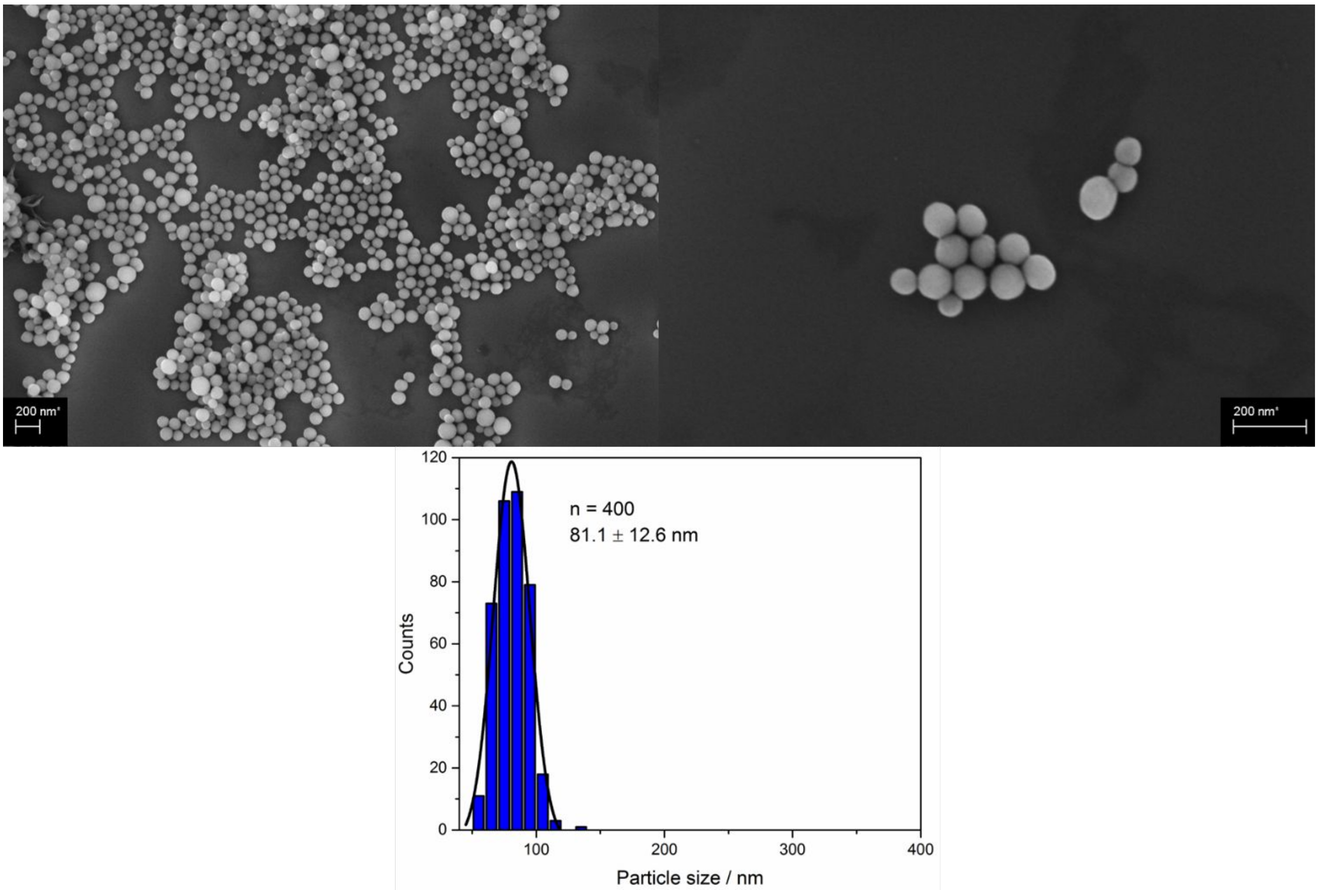

Fig. S2. SEM image of the silica nanoparticles NP-80.

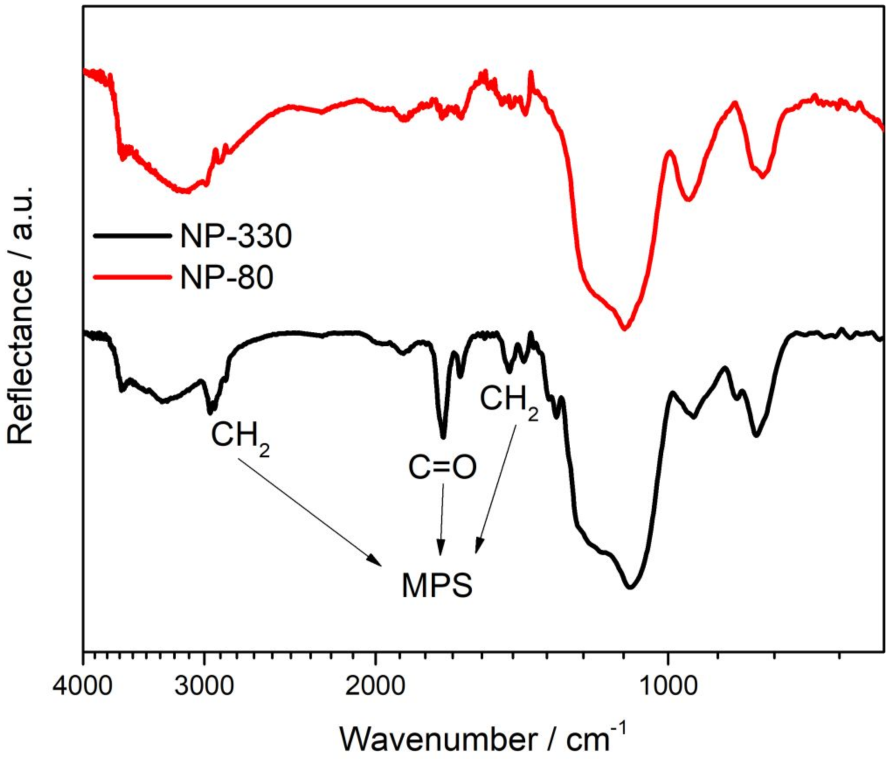

Fig. S3. Infrared spectra of SiO_2 nanoparticles synthesized by the Stöber method and functionalized with MPS.

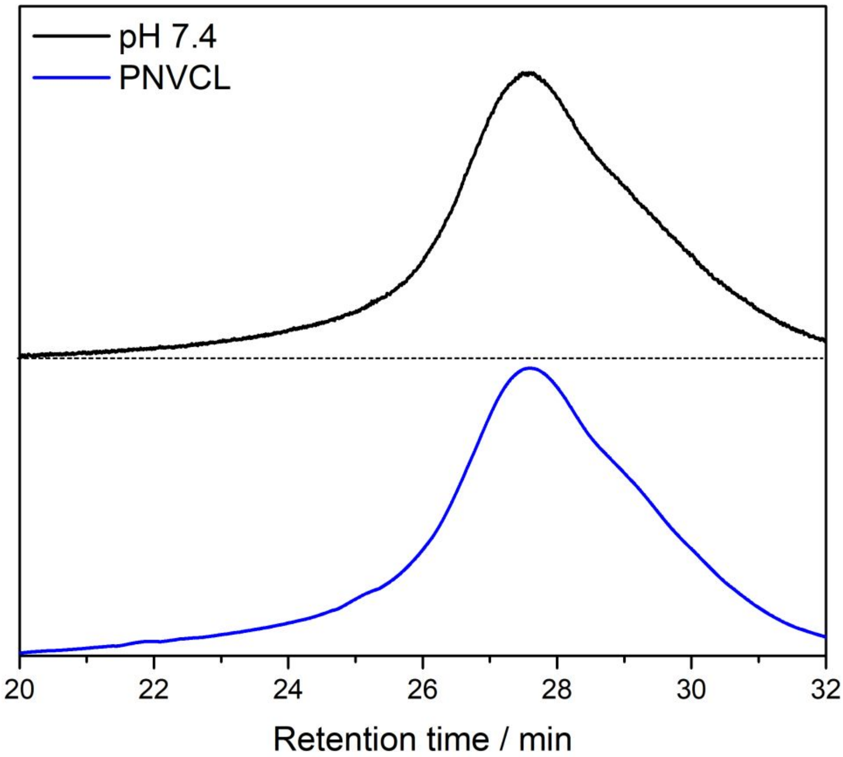

Fig. S4. Gel permeation chromatography (GPC) chromatograms of PNVCL before and after the erosion test.

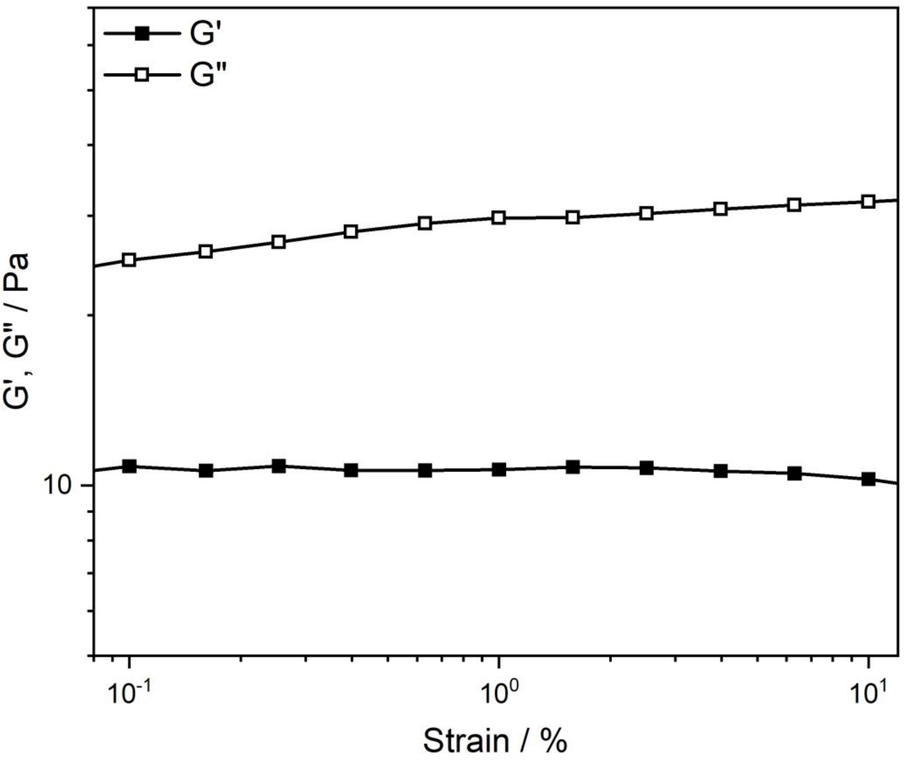

Fig. S5. Amplitude sweep test of 20 wt% solution of PNVCL at 1 Hz and 37 ˚C to determine the linear viscoelastic region (LVR).

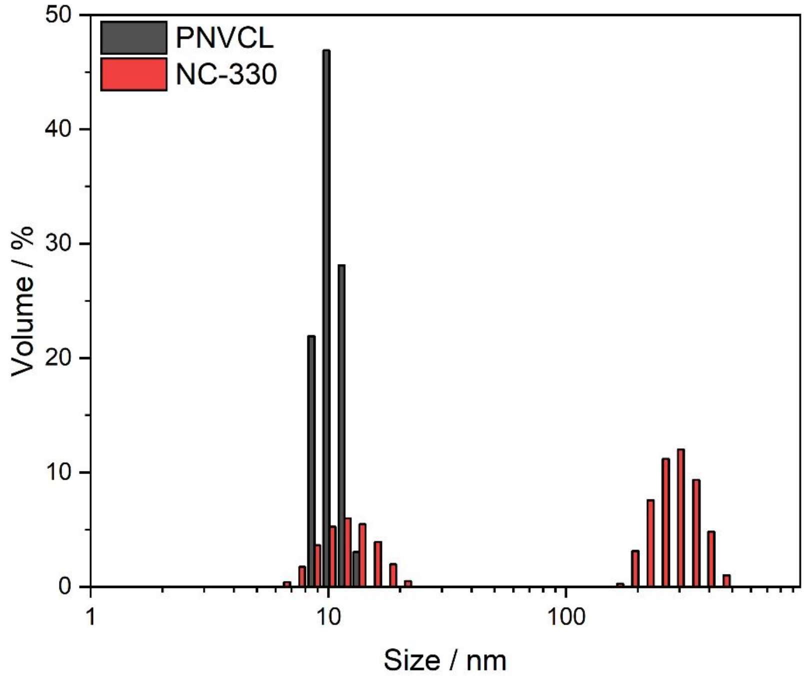

Fig. S6. Analysis of the hydrodynamic diameter for PNVCL and NC-330 measured in the DLS after the erosion tests.
